# Training General Practitioners in Oncology: Lessons Learned From a Cross-Sectional Survey of GPOs in Canada

**DOI:** 10.1200/GO.22.00421

**Published:** 2023-05-04

**Authors:** Bishal Gyawali, Laura Mae Carson, Sian Shuel, Anna Nathalie Wilkinson, Heather Ostic, Colleen Savage, Scott Berry

**Affiliations:** ^1^ Department of Oncology, Queen's University, Kingston, Ontario, Canada; ^2^ Division of Cancer Care and Epidemiology, Queen's Cancer Research Institute, Kingston, Ontario, Canada; ^3^ Department of Public Health Sciences, Queen's University, Kingston, Ontario, Canada; ^4^ Primary Care Program, BC Cancer, Vancouver, British Columbia, Canada; ^5^ Department of Family Medicine, University of Ottawa, Ottawa, Ontario, Canada

## Abstract

Many countries face a significant shortage of medical oncologists. To mitigate this problem, some countries, including Canada, have established training programs for general practitioners in oncology (GPOs), which train family physicians (FPs) in the fundamentals of cancer care. This type of GPO training model may be useful in other countries facing similar challenges. Therefore, Canadian GPOs were surveyed to learn from their experiences and inform the development of similar programs in other countries.

**METHODS:**

A survey was designed and administered to Canadian GPOs to understand the methods and outcomes of GPO training and practice in the Canadian context. The survey was active from July 2021 to April 2022. Participants were recruited through personal and provincial networks and an email list provided by the Canadian GPO network.

**RESULTS:**

The survey received 37 responses for an estimated response rate of 18%. Although only 38% of respondents indicated that family medicine training sufficiently prepared them to care for patients with cancer, 90% indicated that GPO training did. Clinics with oncologists were found to be the most effective mode of learning, followed by small group learning and online education. Critical knowledge domains and skills most relevant for GPO training were identified as the treatment of side effects, symptom management, palliative care, and breaking bad news.

**CONCLUSION:**

Participants in this survey felt that a dedicated GPO training program offered value beyond family medicine residency in preparing providers to adequately care for patients with cancer. GPO training can be done effectively through virtual and hybrid content delivery. Critical knowledge domains and skills identified as the most important in this survey may be valuable for other groups and nations implementing similar training programs to increase their oncology workforce.

## INTRODUCTION

Several countries face the challenge of a growing population of patients with cancer but a significant shortage of cancer care providers, including medical oncologists to care for them.^1,2^ Training a medical student to become a medical oncologist to care for patients with cancer is resource and time intensive. To mitigate this challenge, some countries, including Canada, have taken the innovative approach of training general practitioners in oncology (GPOs). In this approach, family physicians (FPs) are provided with specialized, focused training in the fundamentals of cancer care.

**CONTEXT**

**Key Objective**
To collect information on the training and work experience of Canadian general practitioners in oncology (GPOs) to inform GPO curriculum development elsewhere.
**Knowledge Generated**
A survey of 37 Canadian General Practitioners in Oncology revealed that 90% of respondents felt GPO training prepared them to care for patients with cancer. The modes of training that were most effective included clinics with oncologists, small group learning, and online education. Treatment of side effects, symptom management, palliative care, and breaking bad news were considered the most relevant domains for GPO practice.
**Relevance**
GPOs considered themselves better prepared to care for patients with cancer after GPO training program versus after family medicine residency alone. The results of this survey may offer valuable insights for the development of GPO training programs in other settings.

After training, GPOs can participate in cancer care in both task-shifting and task-sharing models. Under the task-shifting model, GPOs work in collaboration with medical oncologists in larger centers to provide cancer care in locations where medical oncologists are unavailable.^1^ Under the task-sharing model, they work with medical oncologists to provide cancer care so that one oncologist can care for a larger number of patients than would usually be possible.^3^

Over 40 years ago, Canada began using GPOs to deliver cancer care. Now, the country has a dedicated professional national association of GPOs called the Canadian Association of General Practitioners in Oncology (CAGPO).^4^ CAGPO is a membership-based organization that provides educational resources for Canadian GPOs and scholarship funding for trainees, and hosts an annual national conference. The mission of the organization is to unify GPOs, promote communication among them, and to act and speak as the recognized authority on behalf of GPOs and their interests.^5^ In Canada, where many rural residents live a significant distance from a large cancer center, the integration of GPOs has increased access to cancer care.^6^ In addition to offering a more succinct and accessible training program, GPO training programs increase the cancer care workforce in a given region, ultimately making high-quality cancer care more widely available.

In Canada, GPO training programs are established independently by universities and health facilities in different provinces, and various training opportunities are available to general practitioners. In a previous systematic review, we have identified six different GPO training programs in Canada, five of which had a formal curriculum and ranged between 1 month and 1 year in duration.^7^ These existing GPO training programs in Canada are heterogeneous and are coordinated at the provincial rather than national level. The training can vary in duration and content, and both formal and informal training opportunities exist.^7^ Although some training programs in Canada integrate GPO training under the third year of family medicine residency program, some others offer it under continued medical education format.

Many low- and middle-income countries (LMICs) continue to face shortages of cancer care providers, and some LMICs have already responded to this challenge by establishing task shifting and sharing models of care.^1^ One country facing this challenge is Nepal, a small South Asian nation with a population of more than 30 million and a growing cancer burden.^8,9^ Our team previously conducted a survey among general practitioners (GPs) in Nepal and found a strong need and an enthusiastic interest for a GPO training program in Nepal.^12^ As a foundation for developing the curriculum for a training program in Nepal, we conducted a survey of GPOs working in Canada to learn from their training and work experiences.

## METHODS

A survey was designed and iteratively developed by the research team with input from multiple authors and colleagues that included several GPOs and medical oncologists from Canada and underwent internal testing among the GPOs working at Kingston General Hospital before distribution. It was then distributed to Canadian GPOs using Research Electronic Data Capture (REDCap), a secure web-based software platform hosted at Queen's University in Kingston, Ontario, Canada. The survey was approved by the Queen's University Health Sciences and Affiliated Teaching Hospitals Research Ethics Board (HSREB).

The survey contained various response types, including multiple-choice, ordinal scale questions, and interval scale questions (Data Supplement [Supplementary Appendix]). The survey consisted of the following sections: Experience with patients with cancer during FP and GPO trainings, Methods of education about cancer care in GPO programs, Current scope of GPO practice, Oncology related services available to GPOs, and Respondent demographics. The survey was anonymous and did not ask for any personal identifying information from the participants.

The survey was opened in July 2021 and remained active until April 2022. Participants were recruited through personal and provincial network newsletters and an email communication through the CAGPO network to its members. Survey respondents were FPs who had worked or were currently working as GPOs in Canada. The participants were aware that the purpose of the survey was to learn from Canadian GPO's experience as a part of the project to inform the development of a GPO training curriculum for Nepal.

Responses were recorded in REDCap and analyzed in Microsoft Excel. Descriptive statistics are reported in this study.

## RESULTS

### Demographics

Of the 207 GPOs in Canada who received our invitation to participate in the survey, 37 responded for an estimated response rate of 18%. Table 1 shows the demographic information of the respondents. The majority of respondents were female (78%), and most (67%) were between the age of 45 and 64 years. In our sample, nearly 30% of respondents had been practicing as a GPO for <6 years, and 8% had been practicing as a GPO for >20 years. Most respondents (83%) had attended medical school in Canada. Most GPOs (58%) practiced in a large population center with more than 100,000 inhabitants, and only 14% served in small centers with <30,000 inhabitants. Nearly 70% of participants held a university appointment at the time of the survey, and 56% practiced in a university-affiliated hospital. Most respondents (78%) were still practicing as GPOs.

**TABLE 1:**
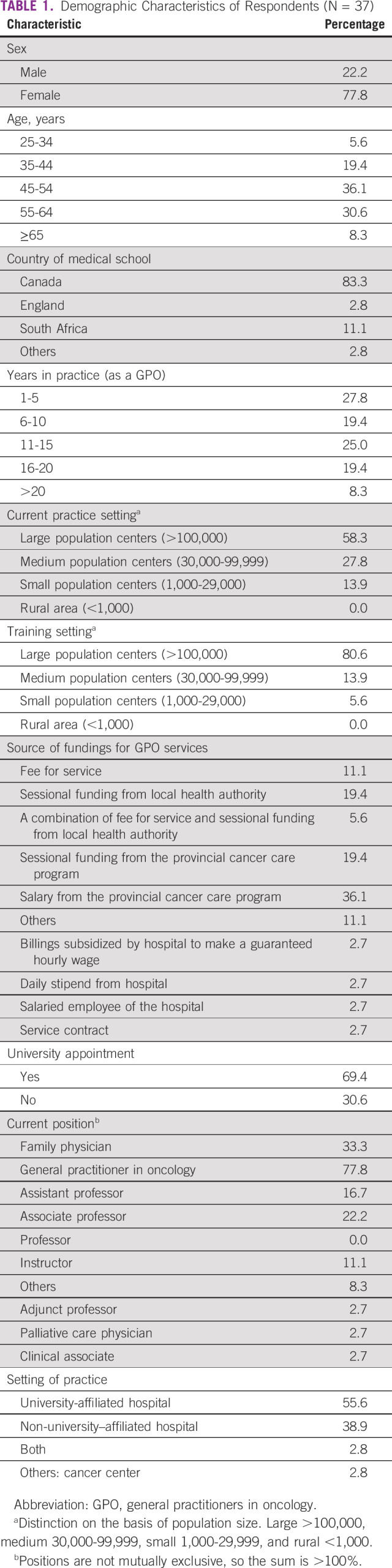
Demographic Characteristics of Respondents (N = 37)

The survey showed that respondents received payments for their oncologic services from a range of sources. More than half of GPOs received compensation from a provincial cancer care program via either salary (36%) or sessional funding (19%) and another 19% received sessional funding from a local health authority.

### Training Experience

#### Oncology training experience during family medicine residency.

Respondents were asked to describe their oncology training experience during their family medicine residency. Approximately one third of respondents (13) had chosen to participate in an oncology rotation during their family medicine training of which only one reported surgical oncology being available as a rotation option, and none of the respondents indicated that gynecologic oncology was an available rotation. The oncology rotation that was most widely available was medical oncology, with 10 participants indicating its availability. Additional rotations in general practice oncology (23%), radiation oncology (31%), hemato-oncology (15%), and surgical oncology (8%) were available during residency. During family medicine training, most respondents (53.8%) had an equal amount of in-patient and out-patient clinical interactions with patients with cancer.

**FIG 1:**
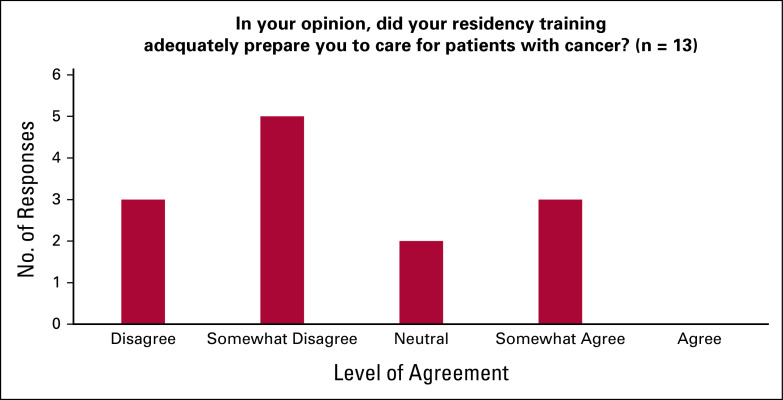
Perceived preparation to care for patients with cancer from family medicine residency training.

**FIG 2:**
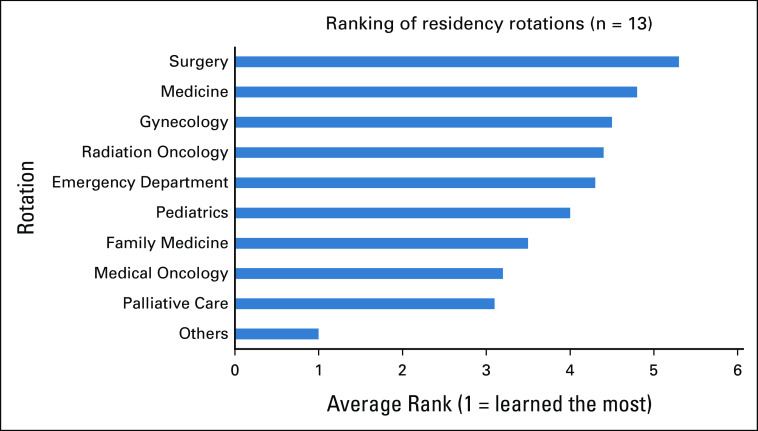
Rank the following rotations in terms of where you learned the most about caring for patients with cancer during your family medicine training.

Of the 13 respondents, none fully agreed and only three (38%) somewhat agreed that their family medicine residency training adequately prepared them to care for patients with cancer (Fig 1). When asked which residency rotations taught respondents the most about caring for patients with cancer, the top-ranked responses were palliative care, medical oncology, and family medicine (Fig 2).

#### GPO training experience.

Forty-three percent of respondents (n = 16) received formal GPO training. Nearly two thirds of respondents (n = 10) participated in a GPO training program that was 2 months in length, and an equal proportion of them engaged in full-time vs. part-time GPO training. Only one (6%) had training of 1 year or longer.

The most frequently available oncology rotations during GPO training were out-patient medical oncology (94%), hematologic oncology (63%), and radiation oncology (63%). In contrast, the rotations that were reported as available by the lowest percentage of respondents were psychosocial oncology (13%), surgical oncology (13%), and pediatric oncology (19%). In contrast to family medicine training, clinical interaction with patients during GPO training was mostly out-patient (69%) or all out-patient (25%).

**FIG 3:**
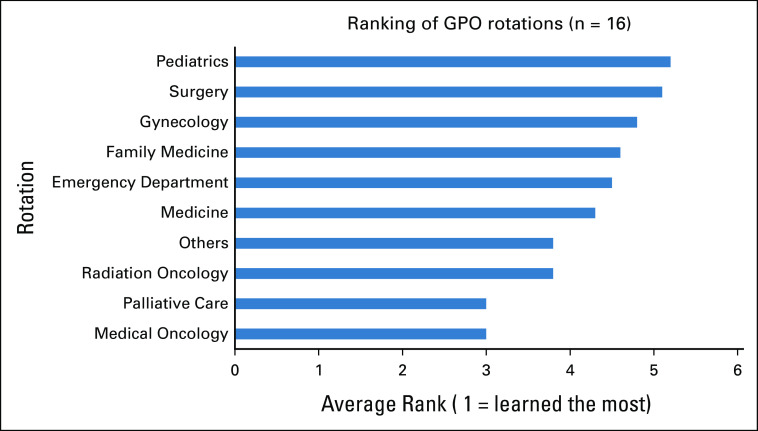
During which rotations did you learn the most about caring for patients with cancer during your GPO training? GPO, general pratitioners in oncology.

**FIG 4:**
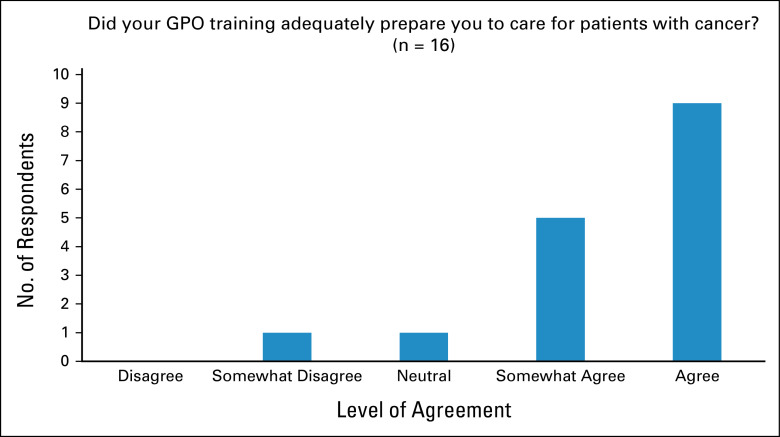
Perceived preparation from GPO training. GPO, general practitioners in oncology.

When asked to rank rotations in terms of where the most was learned about caring for patients with cancer, the rotations with the top three rankings were palliative care, medical oncology, and radiation oncology (Fig 3). In contrast to the responses related to family residency training, nearly 90% agreed that the GPO training program had adequately prepared them to care for patients with cancer, and no respondents disagreed (Fig 4).

In terms of content delivery, respondents indicated a wide range of teaching methodologies during their GPO training. The most common teaching modalities were in-clinic training with oncologists (100%), didactic lectures by oncologists (88%), and self-directed online/web-based learning (87%). The least common modes were didactic lectures by GPOs (31%), small group learning (58%), and in clinic with GPOs (59%). When asked about didactic lectures given by FPs, 10 respondents (90%) indicated that these were effective in learning about caring for patients with cancer. The results were similar when asked about didactic lectures given by oncologists. All respondents (100%) reported clinics with oncologists to be an effective teaching method. Of those who experienced online learning, no respondents felt that it was ineffective. Clinics also proved to be a valuable tool for learners, with 80% (n = 33) of respondents finding clinics with GPOs and 60% (n = 19) of respondents finding clinics with oncologists very effective in learning about caring for patients with cancer.

Of those GPOs who did not receive formal training (n = 21), the delivery methods thought to be the most useful were clinical rotation with oncologists (90%), followed by clinical rotation with GPOs (86%) and then online learning (76%).

### Practice

**FIG 5:**
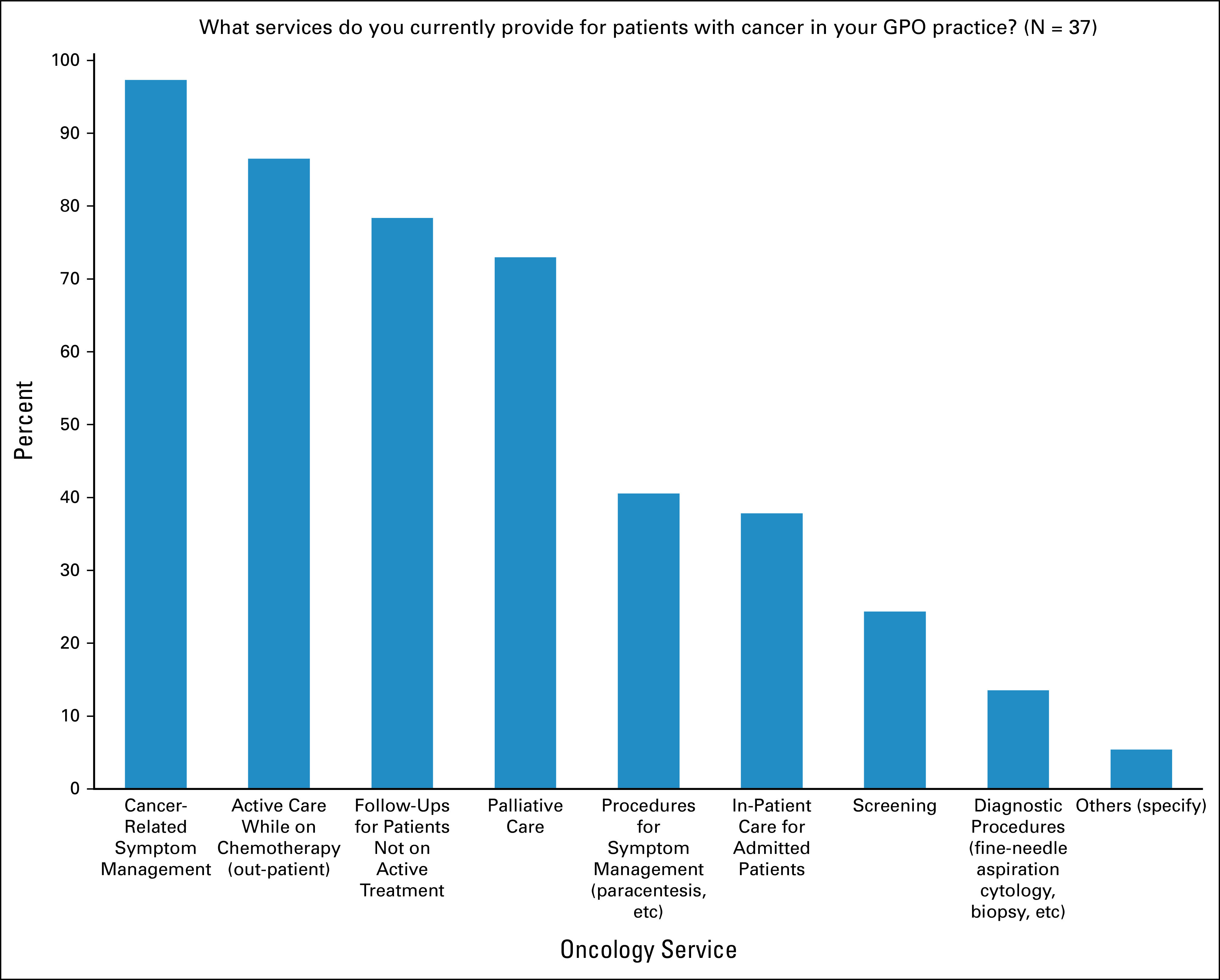
Service provision in GPO practice. GPO, general practitioner in oncology.

Data were collected on the current clinical practice of respondents. Nearly 70% indicated that their current practice as a GPO was mostly out-patient. Most respondents (81%) cared for between 6-15 patients with cancer during a typical out-patient clinic day. In their role as a GPO, 73% of respondents reported caring for an average of one to five patients with cancer per day in an in-patient setting. Participants were asked which tumor types were most commonly presented at their clinic. Of the 37 respondents, 12 indicated lung cancer as most common, followed by 10 for breast cancer and six for hematological malignancies. No respondents indicated cervical, prostate, or skin as the most common tumor type presenting at their clinic. When asked about service provision for patients with cancer in their GPO practices, the four most commonly provided services were cancer-related symptom management, active out-patient care while on chemotherapy, follow-ups for patients not on active treatment, and palliative care. The three least provided services were diagnostic procedures (eg, fine needle aspiration cytology, biopsy, etc), screening, and in-patient care for admitted patients (Fig 5).

In terms of the composition of their professional teams, all worked with nurses, and most had pharmacists (97.3%) and medical oncologists (83.8%) on their teams. Least frequently listed as team members were psychologists (27.0%), occupational therapists (29.7%), hematologists (54.1%), and surgical oncologists (54.1%).

Respondents were asked to reflect on what procedural skills a GPO should be responsible for in practice. Most important skills were managing radiation and chemotherapy toxicities (97% indicated as important), cancer-related symptom management (94% indicated as important), and pain control (92%). Peripherally inserted central catheter line insertion received the lowest score, with only 3% of respondents indicating it as an important skill.

With regards to clinical and communication skills, the most important ones were managing common treatment side effects, managing pain and other symptoms of cancer, and breaking bad news. The least important skill was approach to patient with increased risk of cancer. In terms of knowledge domains important for a GPO's practice, the most important ones were oncology emergencies and the treatment of side effects of cancer treatment, and the least important were the role of nutrition and diet, screening for common cancers, and the epidemiology of common cancers.

**FIG 6:**
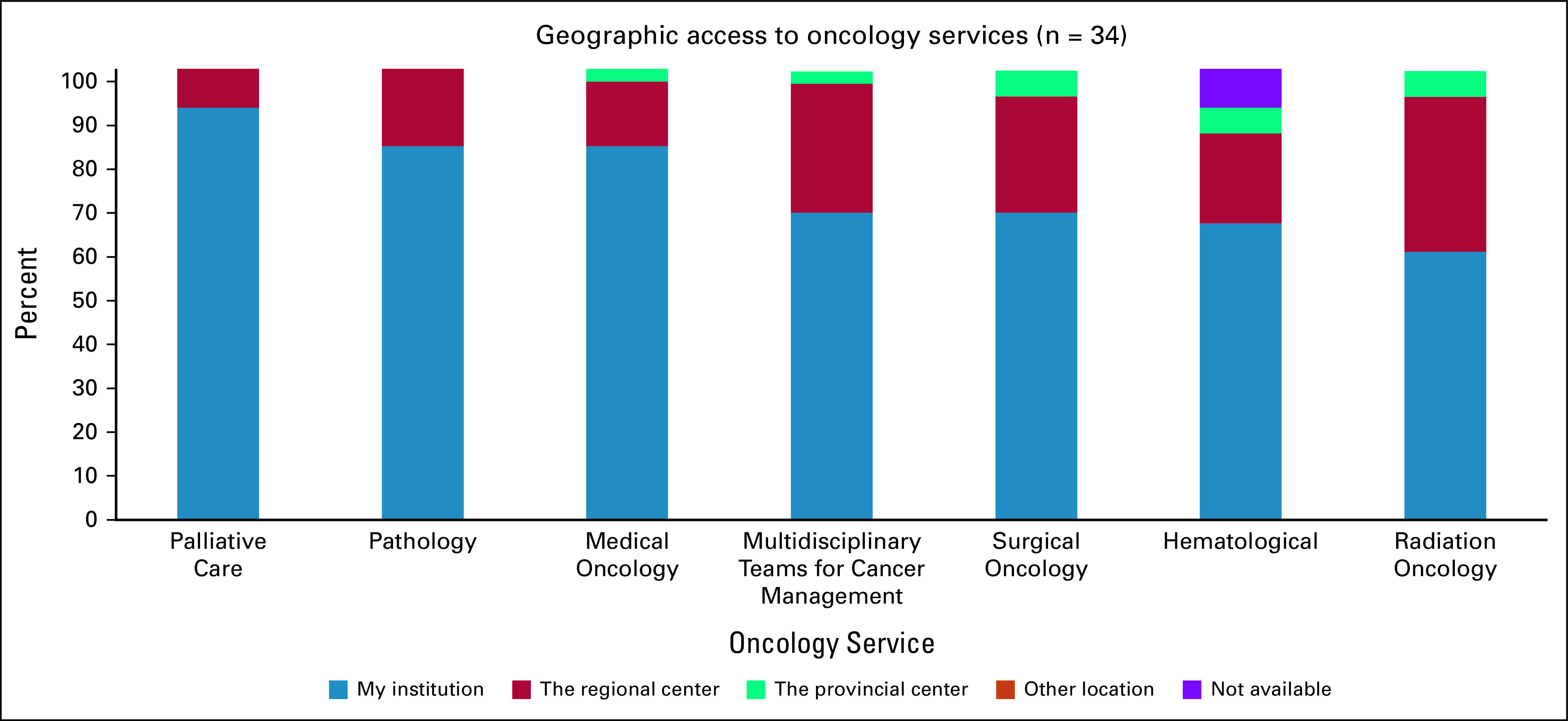
Geographic access to oncology services.

Survey participants also reported on their local access to oncology services. The services most available within the institution of the respondents were palliative care, pathology, and medical oncology. The services that were least readily available within the respondents' institutions were radiation oncology, hematology, surgical oncology, and multidisciplinary teams for cancer management (Fig 6).

## DISCUSSION

In this survey, our findings have provided us with unique insights into the experiences of Canadian GPOs. First, family medicine training was shown to have insufficiently prepared respondents to care for patients with cancer, highlighting the need for a specialized oncology training program for GPOs and the need to include further cancer care education in family medicine training. In both residency and GPO training, palliative care and medical oncology were two critical rotations to deliver value in learning how to care for patients with cancer. These two services were also found to have the greatest availability at respondents' institutions. Cancer-related symptom management and active care for patients receiving chemotherapy were the two services most commonly provided, offering key insights into the role of GPOs in Canada. In contrast to family medicine residency training, a strong majority (90%) of respondents agreed that their GPO training had adequately prepared them to care for patients with cancer. This finding affirms the importance of a GPO training program and highlights its potential for widespread implementation in other settings with a shortage of cancer care providers. Our findings are consistent with those found by Yip et al,^10^ who conducted a needs assessment survey on oncology education for family medicine residents in Canada. The authors concluded that family medicine residency had inadequately prepared most respondents to care for patients with cancer. This cancer training insufficiency has also been found in low- and middle-income settings, including Rwanda.^2^

We previously conducted a survey of Nepali GPs and found a need and interest for GPO training programs in Nepal.^12^ Interestingly, both Nepali GPs and Canadian FPs gained the most experience during medical oncology rotation. However, Nepali GPs were exposed to cancer care mostly in inpatient settings, whereas the Canadian FPs were trained in mostly outpatient settings. Unfortunately, in both Nepal and in Canada, they reported that the oncology experience during their GP and family medicine training inadequately prepared them to care for patients with cancer. Fortunately in contrast, 90% of GPOs agreed that their GPO training program did adequately prepare them to care for patients with cancer.

Several differences in the practice settings, patterns, and expectations were evident between the contexts of Canada and Nepal. Although lung and breast were the most common cancers cared for by GPOs in Nepal and in Canada, the most commonly performed services by GPs in Nepal were cervical cancer screening, palliative care, and screening tests. In contrast, the most commonly performed services by GPOs in Canada were symptom management, active care while on treatment, and follow-up care while not on active treatment. Only 24% of the GPOs in Canada reported performing screening tests for patients, and this may reflect the fact that screening is already routinely provided by FPs. In addition, although Nepali GPs considered skills such as fine-needle aspiration cytology, bone marrow biopsy, and delivering end-of-life care as important skills for GPO training in Nepal, Canadian GPOs considered these skills as the least important. In addition, when asked which knowledge domains were most important for a GPO, screening was found to be most important in Nepal, whereas treatment of side effects of cancer treatment and oncology emergencies were the top responses in Canada, and again, screening ranked the lowest. This latter finding is expected as the task of screening falls primarily to FPs rather than GPOs in Canada. In Nepal, currently there is no clear demarcation of labor regarding which physicians are responsible for cancer screening.

These observations will be valuable for designing and implementing GPO training programs in Nepal. GPO training programs seem to adequately prepare primary care providers to care for patients with cancer, suggesting their utility in settings where cancer care specialist providers are in short supply. The brief duration of additional training increases feasibility and could appeal to health care providers who do not wish to pursue full oncology specialization. Moreover, the short duration means more providers can obtain these critical skills efficiently, allowing more rapid improvements in expanding a cancer workforce. Although the Canadian GPOs typically received training for only a few months, the formal 1-year GPO training program underway in Nepal will provide the opportunity for additional, locally tailored training incorporating the local needs, expectations, regulatory requirements, career opportunities, and feasibility.

The high proportion of respondents who received some component of self-directed or web-based learning in their training suggests the potential for flexibility for the delivery of training content. This is beneficial in terms of expanding training programs to regions where the capacity for local instructors may be limited. Virtual training, such as those available via CAGPO,^5^ could engage greater cross-border collaboration, allowing learners to receive instruction from experts in the field, regardless of location. Our findings also suggest that out-patient clinical rotations are the most useful modality of training in addition to didactic lectures.

In our survey, 70% of GPOs had university appointments and 56% practiced in a university hospital. Although we do not have these metrics for the overall Canadian GPOs, the fact that the majority of respondents in our survey work in academic setting reflects the academic value of GPOs in addition to their primary goal of expanding availability of high-quality cancer care. In addition, several remote locations in Canada are affiliated with universities and physicians hold university appointments despite working in rural areas. Finally, GPOs in an academic setting may also be more likely to respond to surveys.

Our study has several limitations. One limitation is the lack of knowledge of the distribution of GPOs in Canada. The current number of GPOs in Canada and their demographics is unknown. In addition, the sample size of this survey is relatively small (N = 37), and lack of knowledge on the number and distribution of GPOs in Canada makes determining the generalizability of our data difficult. However, sex and age distributions of the sample were noted to be consistent with the national cohort where 62% of Canadian GPOs were females and half were younger than 50 years.^11^ Furthermore, the lack of information from the rural GPO's perspective may limit the applicability of results to nonurban settings where elements of service provision could be more challenging. Although GPOs in Canada are predominantly involved in a task-sharing model given that they mainly work in urban cancer centers with oncologists, the GPOs in Nepal will be expected to work primarily in a task-shifting model where they provide care for patients with cancer in remote locations. The selection of relevant skills or the distribution of tumor type could vary in different regions on the basis of the specific population and their needs. However, for health systems in need of an increased cancer care workforce in urban areas, our data from Canadian GPOs are likely applicable. Recall bias may have been a factor for those who received training many years ago, potentially introducing inaccurate training details. The study also had a small sample size, which was unfortunately challenging to avoid given that the number of GPOs produced every year in Canada is still small. However, our findings suggest that there is a substantial benefit to developing a dedicated GPO training program. The implementation of a coordinated national program in Nepal may provide future insight for Canadians or others interested in expanding their current training model to a systematic and harmonized national approach.

In conclusion, our findings provide insight into the Canadian GPO training and practice experience. Dedicated GPO training programs offer a unique opportunity to efficiently teach health care providers how to adequately care for patients with cancer beyond their family medicine residency training through virtual and hybrid content delivery. Furthermore, our survey identified critical knowledge domains and skills most relevant for GPO training, including treatment of side effects, symptom management, palliative care, and breaking bad news. These results may be valuable for other nations implementing similar training programs in the hopes of increasing their oncology workforce to better care for patients with cancer.
